# Exposure to maternal obesity programs sex differences in pancreatic islets of the offspring in mice

**DOI:** 10.1007/s00125-019-05037-y

**Published:** 2019-11-26

**Authors:** Lisa M. Nicholas, Mototsugu Nagao, Laura C. Kusinski, Denise S. Fernandez-Twinn, Lena Eliasson, Susan E. Ozanne

**Affiliations:** 1 University of Cambridge Metabolic Research Laboratories and MRC Metabolic Diseases Unit, Wellcome Trust-MRC Institute of Metabolic Science, Level 4, Addenbrooke’s Treatment Centre, Addenbrooke’s Hospital, Cambridge, CB2 0QQ UK; 2 Unit of Islet Cell Exocytosis, Department of Clinical Sciences Malmö, Lund University Diabetes Centre, CRC, Skåne University Hospital, Malmö, Sweden

**Keywords:** Beta cell, Islets, Maternal obesity, Type 2 diabetes

## Abstract

**Aims/hypothesis:**

Obesity during pregnancy increases offspring type 2 diabetes risk. Given that nearly half of women of child-bearing age in many populations are currently overweight/obese, it is key that we improve our understanding of the impact of the in utero/early life environment on offspring islet function. Whilst a number of experimental studies have examined the effect of maternal obesity on offspring islet architecture and/or function, it has not previously been delineated whether these changes are independent of other confounding risk factors such as obesity, postnatal high-fat-feeding and ageing. Thus, we aimed to study the impact of exposure to maternal obesity on offspring islets in young, glucose-tolerant male and female offspring.

**Methods:**

Female C57BL/6J mice were fed ad libitum either chow or obesogenic diet prior to and throughout pregnancy and lactation. Offspring were weaned onto a chow diet and remained on this diet until the end of the study. An IPGTT was performed on male and female offspring at 7 weeks of age. At 8 weeks of age, pancreatic islets were isolated from offspring for measurement of insulin secretion and content, mitochondrial respiration, ATP content, reactive oxygen species levels, beta and alpha cell mass, granule and mitochondrial density (by transmission electron microscopy), and mRNA and protein expression by real-time RT-PCR and Western blotting, respectively.

**Results:**

Glucose tolerance was similar irrespective of maternal diet and offspring sex. However, blood glucose was lower (*p* < 0.001) and plasma insulin higher (*p* < 0.05) in female offspring of obese dams 15 min after glucose administration. This was associated with higher glucose- (*p* < 0.01) and leucine/glutamine-stimulated (*p* < 0.05) insulin secretion in these offspring. Furthermore, there was increased mitochondrial respiration (*p* < 0.01) and density (*p* < 0.05) in female offspring of obese dams compared with same-sex controls. Expression of mitochondrial and nuclear-encoded components of the electron transport chain, L-type Ca^2+^ channel subtypes that play a key role in stimulus-secretion coupling [*Cacna1d* (*p* < 0.05)], and oestrogen receptor α (*p* < 0.05) was also increased in islets from these female offspring of obese dams. Moreover, cleaved caspase-3 expression and BAX:Bcl-2 were decreased (*p* < 0.05) reflecting reduced susceptibility to apoptosis. In contrast, in male offspring, glucose and leucine/glutamine-stimulated insulin secretion was comparable between treatment groups. There was, however, compromised mitochondrial respiration characterised by decreased ATP synthesis-driven respiration (*p* < 0.05) and increased uncoupled respiration (*p* < 0.01), reduced docked insulin granules (*p* < 0.001), decreased *Cacna1c* (*p* < 0.001) and *Cacna1d* (*p* < 0.001) and increased cleaved caspase-3 expression (*p* < 0.05).

**Conclusions/interpretation:**

Maternal obesity programs sex differences in offspring islet function. Islets of female but not male offspring appear to be primed to cope with a nutritionally-rich postnatal environment, which may reflect differences in future type 2 diabetes risk.

**Electronic supplementary material:**

The online version of this article (10.1007/s00125-019-05037-y) contains peer-reviewed but unedited supplementary material, which is available to authorised users.

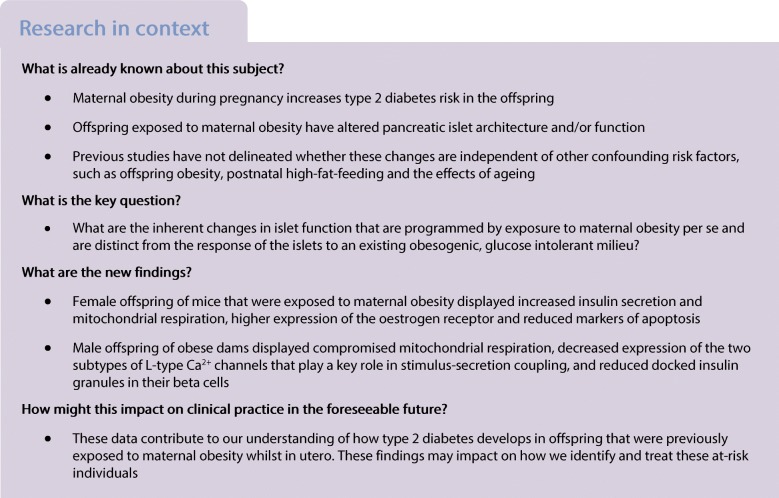



## Introduction

Being overweight or obese is a major risk factor for type 2 diabetes. Importantly, overweight/obesity also has health implications for future generations since obesity during pregnancy increases offspring obesity and type 2 diabetes risk. This was recently highlighted in a study by Lahti-Pulkkinen et al showing that offspring of obese mothers have a 3.5-fold increased risk of type 2 diabetes compared with lean mothers [1]. Abnormalities in beta cell function are critical in delineating the risk of type 2 diabetes because the inability of beta cells to adapt and compensate for peripheral insulin resistance leads to type 2 diabetes pathogenesis [2]. In contrast, sustained beta cell adaptation is capable of preventing type 2 diabetes, even in the face of severe insulin resistance [3].

Given that, in many populations, nearly half of women of child-bearing age are overweight or obese [4], it is important that we improve our understanding of the impact of the in utero and early life environment on offspring islet function. A number of experimental studies have examined the effect of maternal obesity on offspring islet architecture and/or function (reviewed in [5]). Whilst findings of reduced beta cell mass and impaired glucose-stimulated insulin secretion (GSIS) suggest increased type 2 diabetes susceptibility, most previous studies have not delineated whether these changes in islet structure/function are independent of other confounding risk factors such as obesity/increased body weight in the offspring [6–9], postnatal high-fat-feeding [9, 10] and the effects of ageing [8, 10]. Importantly, research by Zambrano et al in 5-week-old rat offspring exposed to maternal obesity suggested that islet function may already be impacted in young life [11].

It is, therefore, key that we identify inherent changes in islet function, which are programmed by exposure to maternal obesity per se and are distinct from the response of the islets to an existing obesogenic, glucose intolerant milieu. This includes investigating the effects on both male and female offspring since sex affects glucose homeostasis and type 2 diabetes risk (reviewed in [12]). For example, using a mouse model of maternal diet-induced obesity, Samuelsson et al showed increased adiposity in both male and female offspring at 6 months of age; however, only male offspring developed type 2 diabetes. This was characterised by higher fasting glucose, lower fasting insulin and decreased pancreatic insulin content. In contrast, these variables were unchanged in female offspring, at the same age [13]. Thus, in the current study, using this same model we characterised pancreatic islet function in younger, metabolically healthy male and female offspring.

## Methods

### Animal model

This research was regulated under the UK Home Office Animals (Scientific Procedures) Act 1986 following ethical review by the University of Cambridge Animal Welfare and Ethical Review Board. The model has been described in detail previously [14]. Briefly, female C57BL/6J mice were randomised to either ad libitum or a standard chow RM1 diet (∼7% simple sugars, 3% fat, 50% polysaccharide, 15% protein [wt/wt], 10.74 kJ/g) or a highly palatable energy-rich obesogenic diet (∼10% simple sugars, 20% animal lard, 28% polysaccharide, 23% protein [wt/wt], 28.43 kJ/g) and sweetened condensed milk (Nestle) (∼16% fat, 33% simple sugars, 15% protein, 13.7 kJ/g) fortified with mineral and vitamin mix (AIN93G), for 6 weeks before mating for first pregnancy. Diets were purchased from Special Dietary Services (Witham, UK). Mice were purchased from Charles River Laboratories (Wilmington, MA, USA). The first litter was culled post weaning. This first pregnancy ensured mice were proven breeders. Mice were then re-mated for a second pregnancy. Dams were maintained on their respective diets throughout both pregnancies and lactation periods. Dams fed an obesogenic diet have around 40% body fat on day one of pregnancy and are hyperinsulinaemic and have impaired glucose tolerance in late gestation [15]. Litters were standardised to six pups on postnatal day two. Offspring from ‘Control’ and ‘Obese’ groups were weaned onto RM1 on postnatal day 21 and remained on this diet until the end of the study. Mice were housed in ventilated cages, 2–3 mice per cage with same-sex littermates, and maintained in a humidity-controlled room with a 12 h light/dark cycle, with free access to food and water. An IPGTT was performed on 7-week-old offspring and the offspring were euthanised at 8 weeks of age. Blood glucose was measured between 07:00 and 08:00 hours on the day of euthanasia from tail blood sample using a blood glucose analyser (AlphaTRAK, Abbot Logistics, the Netherlands). Mice were euthanised by carbon dioxide inhalation. Blood was taken by cardiac puncture for serum insulin analysis. Insulin concentration was determined by ELISA (Mercodia, Sweden).

### IPGTT

Following a 4 h fast, glucose (1 g/kg) was injected into the intraperitoneal cavity. Blood glucose measurements were made using a blood glucose analyser (AlphaTRAK) at 0, 15, 30, 45, 60, 90 and 120 min. Tail blood was also collected at 0, 15 and 30 min into glass micro-haematocrit capillary tubes with sodium heparin (Hirschmann-Laborgeräte, Germany) for plasma insulin analysis. The AUC for glucose was calculated using the trapezoidal rule.

### Pancreatic islet isolation

Islets were isolated as described in [16]. Islets were hand-picked under a stereo microscope and incubated at 37°C overnight in RPMI-1640 medium supplemented with 10% FBS, 100 U/ml penicillin and 100 μg/ml streptomycin sulphate.

### Islet insulin secretion

Groups of 20 islets were incubated in KRB [16] and 2.8 mmol/l glucose for 1 h at 37°C. Next, these groups of islets were incubated at 37°C for 1 h in KRB containing 2.8 mmol/l glucose, 16.7 mmol/l glucose or 10 mmol/l leucine/glutamine.

### Islet insulin content

Islets were washed in PBS mixed with ethanol/hydrochloric acid and sonicated at 4°C. After centrifugation, the supernatant was stored at −20°C until assayed.

### Oxygen consumption assay

Oxygen consumption was measured by the XF24 Extracellular Flux Analyzer (Agilent Technologies, Santa Clara, CA, USA). Groups of 50 islets (in triplicate) were pre-incubated in KRB with 2 mg/ml BSA and 2.8 mmol/l glucose for 30 min at 37°C. Respiration was measured in the presence of 16.7 mmol/l glucose, oligomycin, carbonyl cyanide-4-(trifluoromethoxy)phenylhydrazone (FCCP) and rotenone as previously described [17]. Calculations of basal, glucose-stimulated, ATP-linked and proton leak-linked respiration and coupling efficiency were carried out as previously described [18].

## Detection of reactive oxygen species

Reactive oxygen species (ROS) were detected as previously described [16]. After pre-incubation, islets were loaded with 10 μmol/l 2′,7′-dichlorofluorescin diacetate (DCFDA; Sigma) and incubated at 37°C for 1 h in KRB containing 2.8 mmol/l glucose or 16.7 mmol/l glucose. Fluorescence was measured using a microplate reader with excitation/emission at 495 nm/529 nm.

### Quantification of ATP content

This was performed as previously described [16]. Islets were lysed and the content of ATP at 2.8 or 16.7 mmol/l glucose was assayed using a luciferase-based luminescent assay (Invitrogen, Carlsbad, CA, USA) according to the manufacturer’s instructions.

### Quantification of mRNA expression using quantitative RT-PCR

RNA was extracted (Qiagen, Manchester, UK) and reverse transcribed into cDNA (Fermentas, Waltham, MA, USA). Quantitative RT-PCR (qRT-PCR) was performed using predesigned TaqMan Gene Expression assays or the SYBR Green system (Thermo Fisher Scientific, Carlsbad, CA, USA). mRNA expression was determined by the ΔΔC_t_ method and normalised to *Rplp0* and *Hprt* (females) and *Actb* and *Hprt* (males), expression of which was not influenced by maternal diet. Primer sequences are available in electronic supplementary material (ESM) Table 1.

### Quantification of protein abundance using western blotting

Islets were lysed in RIPA buffer with protease inhibitor cocktail (Sigma, Gillingham, UK), subjected to SDS-PAGE and blotted using antibodies (ESM Table 2). Male and female samples were run on separate gels. Membranes were sectioned and some sections stripped (Restore Western Blot Stripping Buffer, Thermo Fischer Scientific) and re-probed to maximise the amount of data obtained from each western blot. Image Lab software version 5.2.1 (Bio-Rad, Hercules, CA, USA) was used to quantify the density of specific bands. Images of western blots showing statistically significant differences are included in ESM Fig. 1. All blots were made available to *Diabetologia* at the point of submission.

### Mitochondrial DNA content

DNA was extracted (Qiagen) and quantified (Quant-iT PicoGreen dsDNA Reagent, Thermo Fisher Scientific). Equal quantities were amplified by qRT-PCR using primers for nuclear (*Rplp0*) and mitochondrial (*mt-Nd5*) DNA. Mitochondrial (mtDNA) content was determined as described in [19].

### Transmission electron microscopy

Samples were prepared as previously described [16]. Electron micrographs of at least 40 different beta cells from 2–4 mice/sex were taken for each maternal diet group. Granules (large dense-core vesicles) were defined as docked when the centre of the granule was located within 190 nm (a half-length of the mean granule diameter) from the plasma membrane. The distance between the centre of the granule and the plasma membrane was calculated using an in-house software programmed in MatLab 7 (MathWorks, Natick, MA, USA) [20]. Granule density was estimated according to [21] and was normalised to granule diameter assuming spherical geometry [20]. Mitochondrial lesion in beta cells was determined by Photoshop Elements software (Adobe Systems, San Jose, CA, USA). The lesion area was outlined and the size was measured by ImageJ (National Institutes of Health, Bethesda, MD, USA) and expressed as the area density.

### Immunofluorescence

Paraffin-embedded pancreas was exhaustively sectioned (5 μm thickness, 200 μm apart). Sections were incubated with primary antibody against insulin [Dako (Agilent Technologies] and glucagon (Bioss Antibodies, Woburn, MA, USA). The appropriate fluorescent-dye-conjugated secondary antibodies were used for identifying beta and alpha cells (Jackson ImmunoResearch, Ely, UK). Digital images were obtained using Axioscan Z1 Slide Scanner (Zeiss, Germany) and HALO image analysis platform (Indica Labs, Albuquerque, NM, USA) was used to calculate immune-positive and total tissue area. Beta and alpha cell mass was calculated as described in [22].

### Statistical analyses

Investigators were not blind to group assignment and outcome assessment except for analyses of electron micrographs. Calculations were performed in IBM SPSS Statistics 23 (Armonk, NY, USA). ‘*n*’ represents the number of mice from separate litters. Data are presented as mean ± SEM of the indicated number of litters. mRNA expression, protein abundance, mtDNA content and AUC_glucose_ were analysed using unpaired Student’s *t* test. Blood glucose and insulin levels during IPGTT were measured by two-way (repeated measures) ANOVA followed by Bonferroni’s multiple comparisons test (note: each sex was analysed separately). All other data were analysed by two-way ANOVA. A significant interaction indicated sex differences in the impact of maternal obesity. In this case, a simple main effect analysis was performed to isolate the effects of maternal diet on each sex separately. A probability level of 5% (*p* < 0.05) was taken to be significant.

## Results

### Body weight and glucose tolerance are comparable between offspring of control and obese dams at 8 weeks of age irrespective of sex

Body weight, (non-fasted) blood glucose and serum insulin levels were comparable in 8-week-old offspring between maternal diet groups (ESM Table 3). Male offspring, however, were heavier (*p* < 0.05) and had higher serum insulin (*p* < 0.01) (ESM Table 3).

**Fig. 1:**
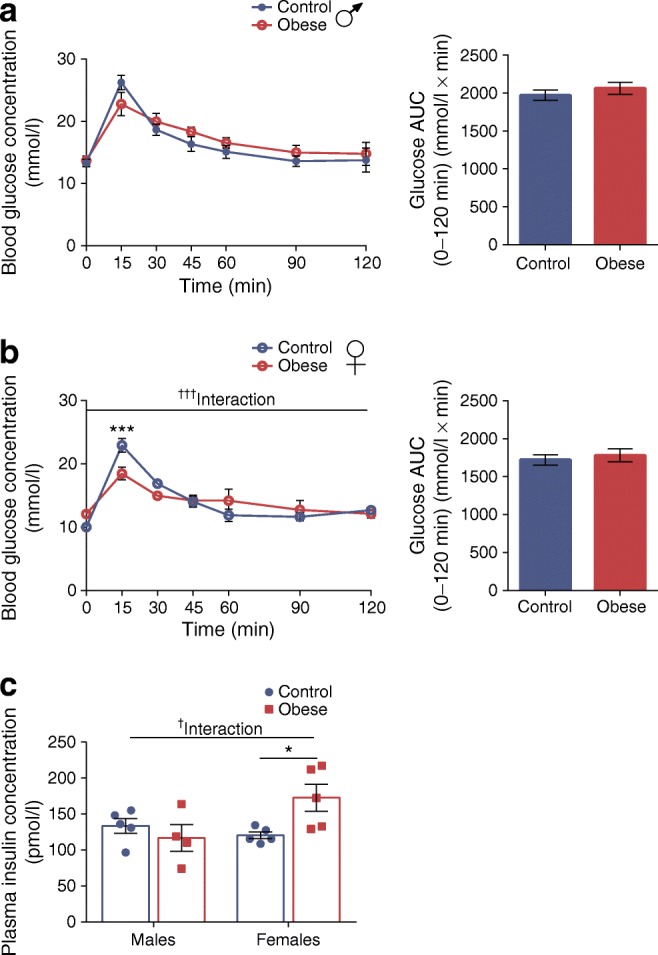
Body weight and glucose tolerance are comparable between offspring of control and obese dams at 8 weeks of age, irrespective of sex. (**a**, **b**) Glucose excursion curves and AUCs during an IPGTT performed on 7-week-old male (**a**) and female (**b**) offspring of control and obese dams after a 4 h fast. (**c**) Plasma insulin concentration at the 15 min time point following glucose administration during an IPGTT in male and female offspring of control and obese dams. (**a**, **b**) Data were analysed by two-way (repeated measures) ANOVA followed by Bonferroni’s multiple comparisons test. (**c**) Data were analysed by two-way ANOVA followed by a simple main effect analysis when a significant interaction was observed. ^†^*p* < 0.05 and ^†††^*p* < 0.001 for interaction; *p < 0.05 and ***p < 0.001 for obese vs control. (**a**, **b**) Males, *n* = 5 mice/group; females, *n* = 5 mice/group; (**c**) males, control: *n* = 5, obese: *n* = 4 mice; females, control: *n* = 5, obese: *n* = 5 mice. ‘*n*’ represents the number of mice from separate litters. All data are mean ± SEM

Glucose tolerance in both sexes, as reflected by AUC, was also similar irrespective of maternal diet (Fig. 1a, b). We observed, however, that blood glucose was lower (*p* < 0.001) and plasma insulin higher (*p* < 0.05) in female offspring of obese dams 15 min after glucose administration compared with controls (Fig. 1b, c).

### GSIS is higher in female but not male offspring that were previously exposed to maternal obesity

**Fig. 2:**
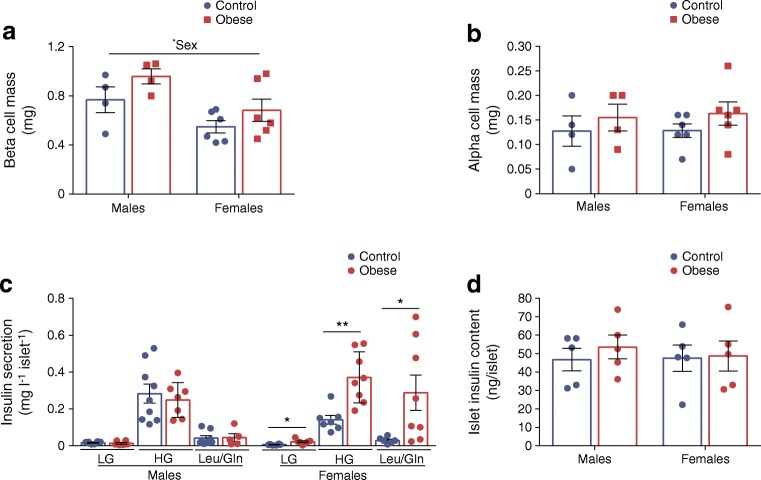
GSIS is higher in female but not male offspring that were previously exposed to maternal obesity. (**a**, **b**) Beta cell (**a**) and alpha cell (**b**) mass in offspring from control and obese dams at 8 weeks of age. (**c**) Insulin secretion stimulated by low glucose (2.8 mmol/l glucose; LG), high glucose (16.7 mmol/l glucose; HG) and 10 mmol/l leucine/glutamine. (**d**) Islet insulin content. Experiments were performed on islets isolated from 8-week-old male and female offspring of control and obese dams. Data were analysed by two-way ANOVA followed by a simple main effect analysis when a significant interaction was observed. There was a significant interaction between maternal diet and offspring sex for data relating to basal (*p* < 0.05), glucose-stimulated (*p* < 0.01) and leucine/glutamine-stimulated (*p* < 0.05) insulin secretion, indicating a sex-specific effect on the outcome measured. *p < 0.05 and **p < 0.01 as shown. (**a**, **b**) Males, *n* = 4 mice/group; females, *n* = 6 mice/group; (**c**) LG: males, control: *n* = 8, obese: *n* = 6 mice; females, control: *n* = 6, obese: *n* = 7 mice; HG: males, control: *n* = 9, obese: *n* = 7 mice; females, control: *n* = 7, obese: *n* = 8 mice; Leu/Gln: males, control: *n* = 8, obese: *n* = 5 mice; females, control: *n* = 6, obese: *n* = 8 mice; (**d**) males and females, *n* = 5 mice/group. ‘*n*’ represents the number of mice from separate litters. All data are mean ± SEM

The amount of insulin secreted by beta cells depends on the mass and function of these cells. Beta cell mass was comparable between maternal diet groups (Fig. 2a). However, in line with having higher serum insulin, male offspring also had increased beta cell mass (*p* < 0.05). This was due to higher absolute pancreas weight (*p* < 0.05) and consequently greater total pancreatic tissue area (*p* < 0.05) with no difference in the relative number of insulin^+^ cells (ESM Fig. 2a–c). In contrast, alpha cell mass was comparable between sexes (Fig. 2b) owing to a lower number of glucagon^+^ cells (*p* < 0.01) in male compared with female offspring (ESM Fig. 2d).

We found differences in beta cell function between male and female offspring of obese dams. Basal (*p* < 0.05), GSIS (*p* < 0.01) and amino acid-stimulated (*p* < 0.05) insulin secretion were increased only in female offspring of obese dams (Fig. 2c). Islet insulin content was not different between groups (Fig. 2d).

### Sex differences in mitochondrial respiration in offspring of obese dams

**Fig. 3:**
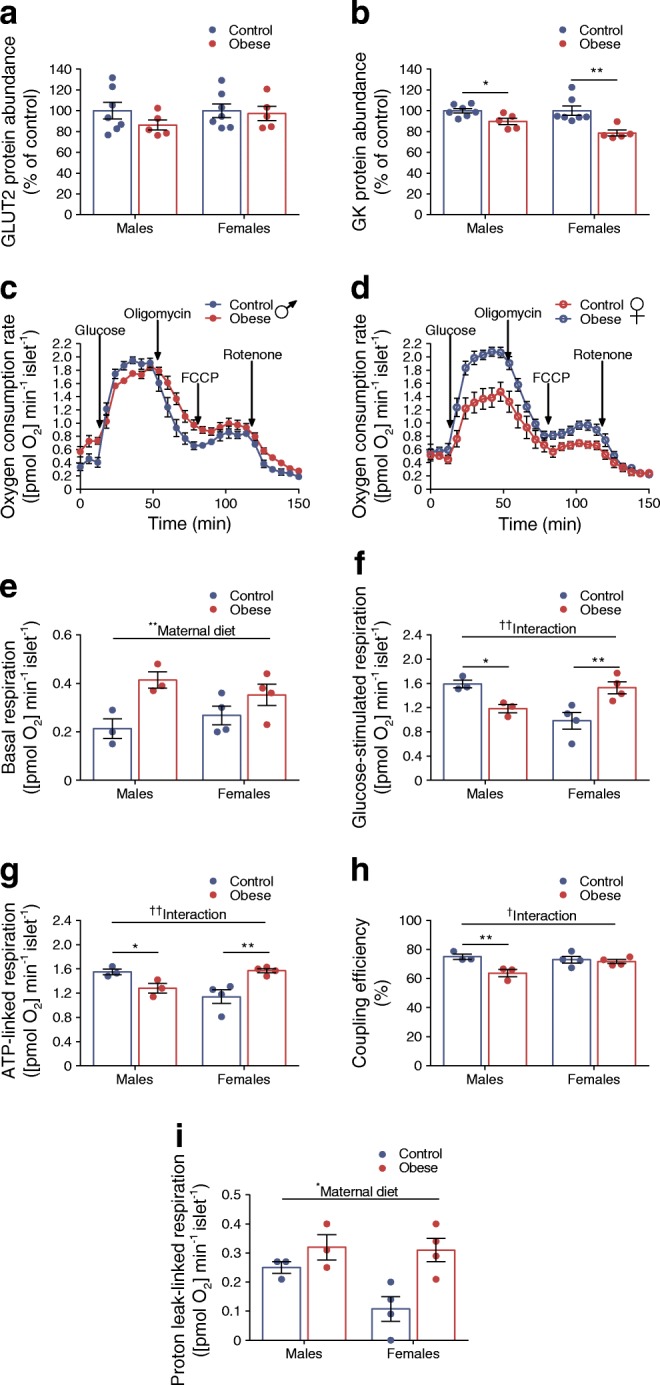
Sex differences in mitochondrial respiration in offspring of obese dams. (**a**, **b**) Western blot analysis of GLUT2 (**a**) and glucokinase (GK) (**b**); images of corresponding western blots are in ESM Fig. 1a, b, respectively. (**c**, **d**) Changes in islet oxygen consumption rate in male (**c**) and female (**d**) offspring of control and obese dams following treatment with 16.7 mmol/l glucose, 4 μg/ml oligomycin, 4 μmol/l FCCP and 5 μmol/l rotenone. (**e**–**i**) Basal (2.8 mmol/l glucose) respiration (**e**), glucose-simulated respiration (16.7 mmol/l glucose) (**f**), ATP-linked respiration (**g**), coupling efficiency (**h**) and proton leak-linked respiration (**i**). Experiments were performed on islets isolated from 8-week-old male and female offspring of control and obese dams. (**a**, **b**) Data were analysed by unpaired Student’s *t* test (control vs obese); (**e**–**i**) data were analysed by two-way ANOVA followed by a simple main effect analysis and a Sidak’s multiple comparisons test when a significant interaction was observed. ^†^*p* < 0.05 and ^††^*p* < 0.01 for interaction; *p < 0.05 and **p < 0.01 as shown. (**a**, **b**) Males and females, control: *n* = 7, obese: *n* = 5 mice; (**c**–**i**) males, *n* = 3 mice/group; females, *n* = 4 mice/group. ‘*n*’ represents the number of mice from separate litters. All data are mean ± SEM

To identify cellular components that may be contributing to enhanced GSIS in female offspring, we quantified GLUT2 protein abundance, the primary glucose transporter in rodent islets [23]. This was not altered by exposure to maternal obesity (Fig. 3a). Subsequent phosphorylation of glucose by glucokinase is the key step controlling glycolytic flux [24]. Glucokinase abundance was reduced in both male (*p* < 0.05) and female (*p* < 0.01) offspring of obese dams (Fig. 3b). This finding was unexpected given that the rate of glycolysis is an important determinant of GSIS from beta cells.

Mitochondrial metabolism couples glucose metabolism to insulin secretion. Since leucine/glutamine are mitochondrial fuels, this suggested that pathways downstream of glucokinase may be activated in these islets. We found sex differences in mitochondrial function in offspring of obese dams. Whilst exposure to maternal obesity resulted in higher basal respiration irrespective of offspring sex (*p* < 0.01) (Fig. 3c–e), glucose-stimulated (relative to basal) respiration was reduced in males (*p* < 0.05) but increased in female (*p* < 0.01) offspring of obese dams (Fig. 3c, d, f).

Respiration rate is mostly composed of ATP-linked and proton leak-linked respiration [25]. Similar to glucose-stimulated respiration, ATP-linked respiration was reduced in male offspring of obese dams (*p* < 0.05) (Fig. 3g). Moreover, coupling efficiency, which estimates the fraction of respiration used to drive ATP synthesis was also reduced in these islets (*p* < 0.01) (Fig. 3h). In contrast, ATP-linked respiration was increased in female offspring of obese dams (*p* < 0.01) (Fig. 3g). Finally, we also found that proton leak-linked respiration was higher in islets from maternal obesity-exposed offspring (*p* < 0.05) (Fig. 3i). This appears to be driven by a relatively large difference between female offspring groups due to lower proton leak-linked respiration in the control group.

### Only female offspring of obese dams have increased expression of mitochondrial and nuclear-encoded components of the electron transport chain

**Fig. 4:**
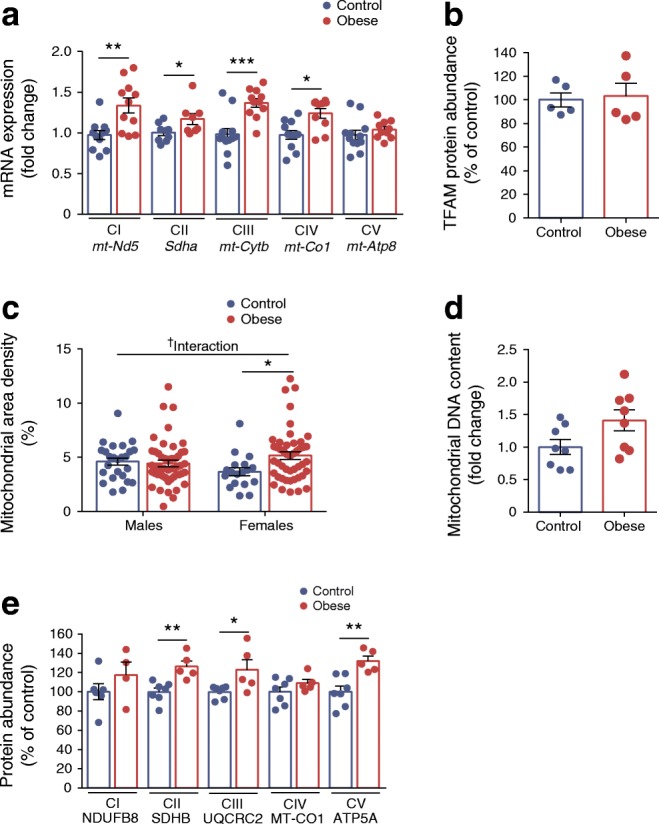
Among female offspring, only the offspring of obese dams have increased expression of mitochondrial and nuclear-encoded components of the electron transport chain. (**a**) qRT-PCR analysis of mRNA expression of mitochondrial (*mt-Nd5*, *mt-Cytb*, *mt-Co1*, *mt-Atp8*) and nuclear (*Sdha*) encoded components of the electron transport chain. (**b**) Western blot analysis of mitochondrial transcription factor A (TFAM); image of corresponding western blot is in ESM Fig. 1c. (**c**) Mitochondrial area density. (**d**) Mitochondrial DNA content. (**e**) Western blot analysis of mitochondrial (MT-CO1) and nuclear (NDUFB8, SDHB, UQCRC2 and ATP5A) encoded components of the electron transport chain; images of corresponding western blots are in ESM Fig. 1d. Experiments relating to (**a**, **b**, **d**, **e**) were performed on islets from female offspring and (**c**) was performed on islets isolated from 8-week-old male and female offspring of control and obese dams. (**a**, **b**, **d**, **e**) Data were analysed independently by unpaired Student’s *t* test (control vs obese). (**c**) Data were analysed by two-way ANOVA followed by a simple main effect analysis when a significant interaction was observed. ^†^*p* < 0.05 for interaction; *p < 0.05, **p < 0.01 and ***p < 0.001 as shown. (**a**) *mt-Nd5*, control: *n* = 11, obese: *n* = 11; *Sdha*, control: *n* = 9, obese: *n* = 8; *mt-Cytb*, control: *n* = 12, obese: *n* = 11; *mt-Co1*, control: *n* = 11, obese: *n* = 10; *Atp8*, control: *n* = 12, obese: *n* = 10 mice; (**b**) *n* = 5 mice/group; (**d**) *n* = 8 mice/group; (**e**) NDUFB8, control: *n* = 6, obese: *n* = 4; SDHB, UQCRC2, MT-CO1 and ATP5A, control: *n* = 7, obese: *n* = 5 mice; ‘*n*’ represents the number of mice from separate litters; in (**c**) the data points shown correspond to the number of beta cells from: males, control: *n* = 2, obese: *n* = 4 mice; females, control: *n* = 2, obese: *n* = 3 mice. All data are mean ± SEM. For (**a**, **b**, **d**, **e)** the equivalent graphs for male offspring are in ESM Fig. 3. CI–V, Complex I–V

Mitochondrial respiration/function is dependent on mtDNA, genes of the respiratory chain complexes that it encodes and its nuclear-encoded constituents. mRNA expression of some mitochondrial-encoded components of the respiratory complexes was significantly increased in maternal obesity-exposed female offspring (Fig. 4a). This was not due to higher levels of mitochondrial transcription factor A (TFAM) (Fig. 4b) but may be partly due to increased number of mitochondria as evidenced by higher mitochondrial area density (*p* < 0.05) (Fig. 4c). There was, however, no statistical difference in mtDNA content (*p* < 0.06; Fig. 4d). In addition to mitochondrial genes, mRNA expression of *Sdha* (Complex II) (*p* < 0.05) (Fig. 4a) and protein abundance of succinate dehydrogenase B (SDHB; Complex II) (*p* < 0.01), ubiquinol-cytochrome C reductase core protein 2 (UQCRC2; Complex III) (*p* < 0.05) and ATP5A (Complex V) (*p* < 0.01), which are nuclear-encoded, were also increased (Fig. 4e).

Our results suggest that higher mitochondrial respiration in female offspring of obese dams could be due to having more mitochondria and higher levels of respiratory complex proteins. The converse is not the case for male offspring (ESM Fig. 3a–d and Fig. 4c).

### Increased ROS in islets from both male and female offspring of obese dams but increased expression of antioxidant enzyme only in female islets

**Fig. 5:**
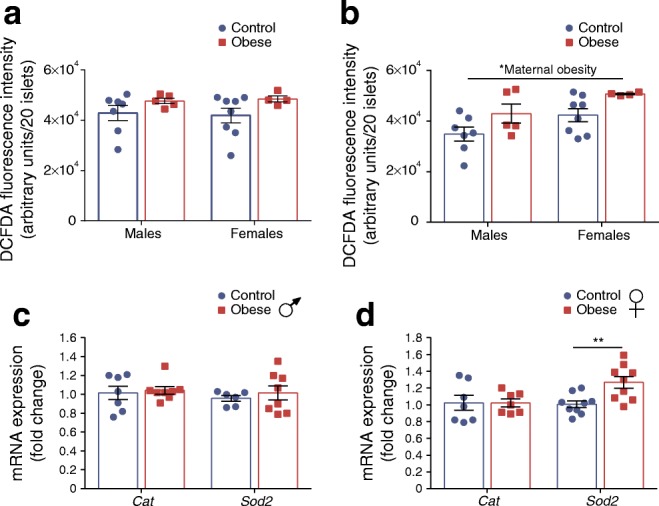
ROS is increased in islets from both male and female offspring of obese dams but expression of antioxidant enzyme is increased only in female islets. (**a**, **b**) ROS levels measured by DCFDA fluorescence intensity following incubation with 2.8 mmol/l glucose (**a**) or 16.7 mmol/l glucose (**b**). (**c**, **d**) qRT-PCR analysis of *Cat* and *Sod2* mRNA expression in male (**c**) and female (**d**) offspring. Experiments were performed on islets isolated from 8-week-old male and female offspring of control and obese dams. (**a**, **b**) Data were analysed by two-way ANOVA followed by a simple main effect analysis when a significant interaction was observed. (**c**, **d**) Data were analysed independently by unpaired Student’s *t* test (control vs obese). *p < 0.05 and **p < 0.01 as shown. (**a**, **b**) Males, control: *n* = 7, obese: *n* = 5 mice; females, control: *n* = 8, obese: *n* = 4 mice; (**c**, **d**) *Cat*: males, control: *n* = 7, obese: *n* = 8 mice; females, control: *n* = 7, obese: *n* = 7 mice; *Sod2*: males, control: *n* = 8, obese: *n* = 6 mice; females, control: *n* = 9, obese: *n* = 9 mice. ‘*n*’ represents the number of mice from separate litters. All data are mean ± SEM

Mitochondria are a major source of intracellular ROS [26]. Basal ROS levels were comparable between offspring groups (Fig. 5a). In response to glucose stimulation, ROS levels were higher in islets of maternal obesity-exposed offspring (*p* < 0.05) (Fig. 5b). Thus, in the case of male offspring of obese dams, ROS levels are elevated even with reduced mitochondrial respiration. Furthermore, female but not male offspring of obese dams displayed increased *Sod2* (*p* < 0.01) (but not *Cat*) mRNA expression (Fig. 5c, d) suggesting that islets from these male offspring may be more vulnerable to oxidative stress.

### Reduction in L-type Ca^2+^ channels and docked insulin granules in beta cells from male offspring that were previously exposed to maternal obesity

**Fig. 6:**
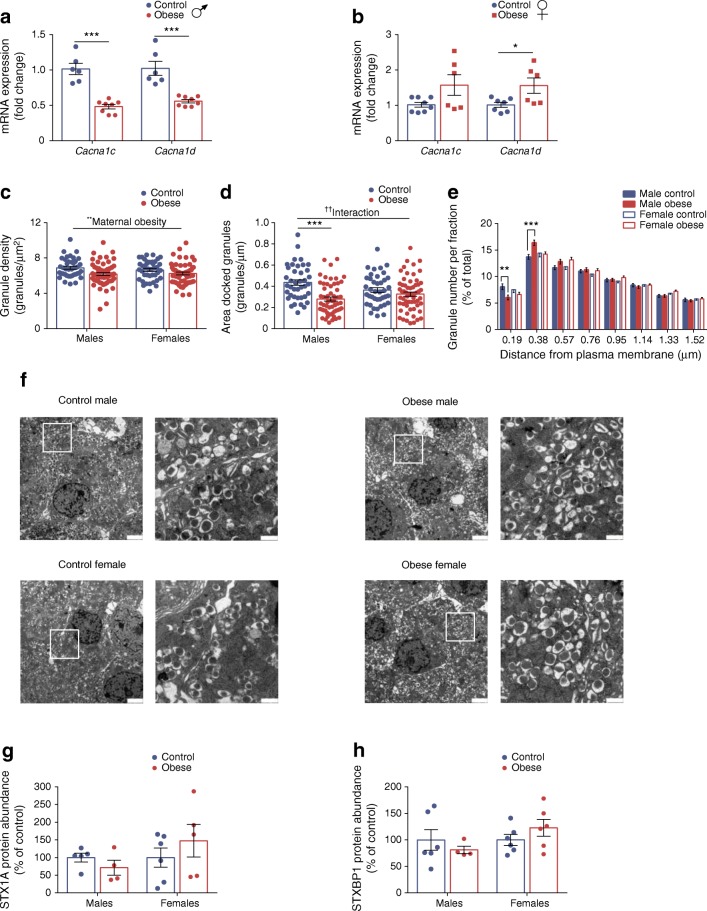
Reduction in L-type Ca^2+^ channels and docked insulin granules in beta cells from male offspring that were previously exposed to maternal obesity. (**a**, **b**) qRT-PCR analysis of *Cacna1c* and *Cacna1d* mRNA expression in male (**a**) and female (**b**) offspring. (**c**–**f**) Transmission electron microscopy analysis of insulin granule density (**c**) and docked granules estimated by the surface density (**d**). Relative distribution of granules at distance fractions from the plasma membrane (**e**). Granules were defined as docked if their distance from the plasma membrane was 0.19 μm, i.e. half the size of the mean granule diameter. Representative transmission electron microscopy micrographs of beta cells from male and female offspring of control and obese dams (**f**). The right-hand image is a magnification of the inset in the left-hand image; scale bars, 2 μm for the left-hand image in each set and 500 nm for the magnification. (**g**, **h**) Western blot analysis of STX1A (**g**) and STXBP1 (**h**); images of corresponding western blots are in ESM Fig. 1e, f, respectively. Experiments were performed on islets isolated from 8-week-old male and female offspring of control and obese dams. (**c**–**e**) Data were analysed by two-way ANOVA followed by a simple main effect analysis when a significant interaction was observed. Analyses based on individual animals (*n* = 2–4 mice/group) showed no statistically significant difference within the groups; thus, cell-based analyses (*n* = 40–59 cells/group) were performed. (**a**, **b**, **g**, **h**) Data were analysed independently by unpaired Student’s *t* test (control vs obese). ^††^*p* < 0.01 for interaction; *p < 0.05, **p < 0.01 and ***p < 0.001 as shown. In (**b**), *p* = 0.05 for *Cacna1c* mRNA expression in female offspring of control vs obese dams. (**a**, **b**) *Cacna1c*: males, control: *n* = 6, obese: *n* = 8 mice; females, control: *n* = 8, obese: *n* = 6 mice; *Cacna1d*: males, control: *n* = 6, obese: *n* = 8 mice; females, control: *n* = 7, obese: *n* = 6 mice; (**g**) males, control: *n* = 5, obese: *n* = 4 mice; females, control: *n* = 6, obese: *n* = 5 mice; (**h**) males, control: *n* = 6, obese: *n* = 4 mice; females, control: *n* = 6, obese: *n* = 6 mice; ‘*n*’ represents the number of mice from separate litters; in (**c**, **d**) the data points shown correspond to the number of beta cells from: males, control: *n* = 2, obese: *n* = 4 mice; females, control: *n* = 2, obese: *n* = 3 mice. All data are mean ± SEM

Activation of L-type voltage-gated Ca^2+^ (Ca_V_1) channels are essential for insulin secretion [27, 28]. Exposure to maternal obesity resulted in sex-specific differences in expression of the two subtypes of Ca_V_1 channels: *Cacna1c* and *Cacna1d* (which encode Ca_V_1.2 and Ca_V_1.3, respectively). *Cacna1d* expression was increased in females (*p* < 0.05) but both *Cacna1c* and *Cacna1d* expression was decreased in male (*p* < 0.001) offspring of obese dams (Fig. 6a, b). Downregulation of these genes has been observed in both human and rodent type 2 diabetic islets [29–31].

Docking of secretory vesicles is required for insulin exocytosis [20, 32]. Insulin granule density was reduced in beta cells of maternal obesity-exposed offspring of both sexes (*p* < 0.01) (Fig. 6c). We found, however, that the area of docked granules was decreased only in male offspring of obese dams (*p* < 0.001) (Fig. 6d). Furthermore, the number of granules in the first fraction beneath the plasma membrane (0.19 μm from the plasma membrane) was reduced (*p* < 0.01) whilst those in the second fraction (0.38 μm from the plasma membrane) were increased (*p* < 0.001) in these mice (Fig. 6e). These results suggest that granules may be stacking in the second fraction (which is considered to be the reserve pool) owing to dysfunctional docking in beta cells of these mice. Expression of syntaxin-1A (STX1A) and syntaxin binding protein 1 (STXBP1) proteins, which are required for docking [33] was unchanged in both male and female offspring (Fig. 6g, h).

### Islets from female offspring of obese dams may be protected from dysfunction by increased oestrogen receptor α and reduced susceptibility to apoptosis

**Fig. 7:**
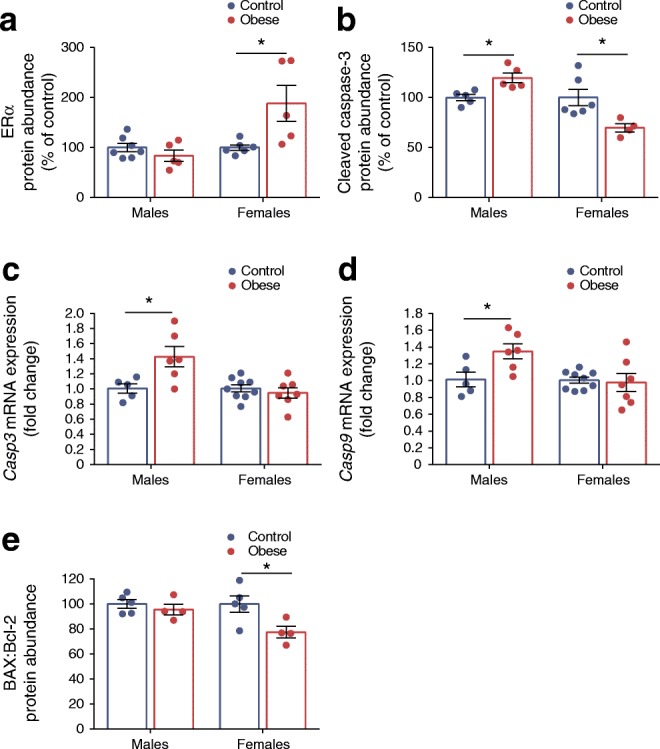
Islets from female offspring of obese dams may be protected from dysfunction by increased ERα and reduced susceptibility to apoptosis. (**a**, **b**) Western blot analysis of ERα (**a**) and cleaved caspase-3 (**b**) protein abundance; images of corresponding western blots are in ESM Fig. 1g, h, respectively. (**c**, **d**) qRT-PCR analysis of *Casp3* (**c**) and *Casp9* (**d**) mRNA expression in male and female offspring. (**e**) Western blot analysis of BAX:Bcl-2 ratio; images of corresponding western blots are in ESM Fig. 1i (BAX) and ESM Fig. 1j (Bcl-2). Experiments were performed on islets isolated from 8-week-old male and female offspring of control and obese dams. Males and females were analysed independently by unpaired Student’s *t* test (control vs obese). *p < 0.05, as shown. (**a**) Males, control: *n* = 7, obese: *n* = 5 mice; females, control: *n* = 6, obese: *n* = 5 mice; (**b**) males, *n* = 5 mice/group; females, control: *n* = 6, obese: *n* = 4 mice; (**c**, **d**) males, control: *n* = 5, obese: *n* = 6 mice; females, control: *n* = 9, obese: *n* = 7 mice; (**e**) BAX: males, *n* = 5 mice/group; females, control: *n* = 5, obese: *n* = 4 mice; Bcl-2: males and females, control: *n* = 5, obese: *n* = 4 mice. ‘*n*’ represents the number of mice from separate litters. All data are mean ± SEM

17β-oestradiol has been shown to protect females from beta cell death and hyperglycaemia via oestrogen receptor α (ERα) [34–36]. In our model, ERα protein abundance was higher in female offspring of obese dams (*p* < 0.05) (Fig. 7a). Furthermore, expression of cleaved (activated) caspase-3, the key mediator of the apoptotic cascade in mammalian cells [37] was decreased (*p* < 0.05) in these offspring (Fig. 7b). In contrast, cleaved caspase-3 abundance and *Casp3* mRNA expression were increased in male offspring of obese dams (*p* < 0.05) (Fig. 7b, c).

The mitochondrial phenotype in this study prompted us to investigate whether the mitochondrial intrinsic apoptosis pathway could be contributing to changes in caspase-3 activity. mRNA expression of *Casp9*, the initiator caspase involved in this pathway, was higher in male offspring of obese dams (*p* < 0.05) (Fig. 7d). Furthermore, the ratio of pro-apoptotic BAX to anti-apoptotic Bcl-2, which can profoundly influence the ability of a cell to respond to an apoptotic signal [38], was lower in female offspring of obese dams (*p* < 0.05) (Fig. 7e). This suggests a downregulation of the mitochondrial apoptosis pathway in these offspring. There were no differences in protein levels of BAX and Bcl-2 between offspring of maternal diet groups (ESM Fig. 4a, b).

## Discussion

Whilst there are known genetic risk factors that may be transferred from mother to child, which explain future obesity and type 2 diabetes risk, the picture is far from complete. Studies in humans that have controlled for shared genetics found that individuals exposed to maternal obesity/gestational diabetes had a greater diabetes and obesity risk compared with unexposed individuals [39–41]. Furthermore, a recent record-linkage study involving 118,201 mothers and their offspring found that being overweight or obese in mothers is associated with increased incidence of offspring type 2 diabetes [1].

Given that in humans a mother’s genes and environment co-exist, rodent models of obesogenic diet-induced obesity across pregnancy and lactation, which resemble the human situation, have been key in our understanding of the non-genetic transfer of metabolic disease risk from mother to offspring. A number of studies have suggested that increased type 2 diabetes susceptibility in developmentally programmed offspring is due to altered pancreatic islet architecture and/or function (reviewed in [5]). The strength of this study is that it is the first to outline the sex-specific changes in islet function in offspring born to obese dams that are present before and are, therefore, independent of offspring obesity, glucose intolerance and ageing. This is important as it allows us to elucidate islet processes that are most vulnerable to dysfunction thus leading to higher type 2 diabetes risk in offspring exposed to maternal obesity.

A limitation of this study is that we did not measure adiposity in male and female offspring at 8 weeks of age. Previous studies in this model have shown, however, that fat mass is comparable in 8-week-old offspring from control and obese dams irrespective of sex [42, 43].

**Fig. 8:**
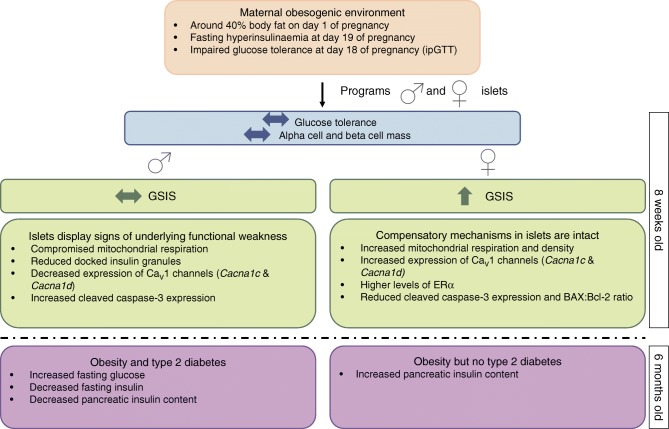
Exposure to maternal obesity programs sex differences in pancreatic islets of the offspring in mice

It has been shown previously, using the same model of maternal diet-induced obesity, that whilst both male and female offspring develop obesity and insulin resistance with age, female offspring of obese dams are less susceptible to type 2 diabetes at 6 months of age [13]. Our findings suggest that islets from female offspring are primed to handle a nutritionally-rich postnatal environment by upregulating mitochondrial respiration and Ca_V_1 expression leading to higher GSIS. Therefore, despite being ‘overworked’ from a young age, these islets appear to have protective mechanisms in place to cope with the demands of increasing adiposity and insulin resistance (Fig. 8). For example, reduced glucokinase expression could act to temper glycolysis in the face of increased mitochondrial metabolism so as to maintain a healthy redox balance [44]. Increased proton leak-linked respiration may also not necessarily be damaging (especially since coupling efficiency is maintained). Mitochondrial superoxide production is steeply dependent on the protonmotive force across the inner mitochondrial membrane. Thus, increased proton leak may act to minimise oxidative damage by moderating the protonmotive force and, therefore, ROS production [45]. Furthermore, increased ERα levels could also have a positive influence on these islets by preserving mitochondrial function [46] and protecting beta cells from apoptosis [34–36]. Indeed, oestrogen-based replacement therapies have been shown to reduce type 2 diabetes incidence in post-menopausal women [47, 48]. As part of future studies, it will be important to determine if serum oestradiol levels are also different between offspring groups. Moreover, it will be key to elucidate islet function and type 2 diabetes risk in post-menopausal, maternal obesity-exposed mice.

In line with the observation that women have lowered risk for type 2 diabetes [49], females appear to be protected from beta cell death in most rodent models of type 2 diabetes [12, 50]. In the case of exposure to maternal diet-induced obesity/high-fat-feeding and future type 2 diabetes risk in the offspring, Yokomizo and colleagues showed that islets from female offspring were better able to compensate and adapt to a high-fat diet (HFD) in postnatal life compared with male offspring [9]. Interestingly, in a study of maternal obesity using the agouti viable yellow (A^vy^) mouse, (aged) female offspring developed glucose intolerance and had reduced GSIS following postnatal HFD compared with males [10]. This is in contrast to findings by Li and colleagues, which showed that the latent predisposition to metabolic disease in offspring of A^vy^ dams, was more prominent in males who developed glucose intolerance and insulin resistance after only 3 weeks of HFD [51]. It should be noted that in contrast to most models of maternal diet-induced obesity, A^vy^ mice are normoglycaemic during pregnancy.

In humans, men are diagnosed with type 2 diabetes at a younger age and at lower levels of adiposity than women [49]. The male sex is also acknowledged as a diabetes risk factor [52]. In this study, islets from maternal obesity-exposed male offspring appear not to fare as well as females (Fig. 8). This manifests as suboptimal mitochondrial respiration. Unlike in females, these changes could not be attributed to changes in mtDNA content or the expression of genes encoding components of the electron transport chain. The mitochondria in islets of male offspring seem particularly vulnerable to insults in utero/early life; male offspring that experienced intrauterine growth restriction followed by postnatal catch-up growth also showed impaired mitochondrial function and increased ROS production prior to the onset of diabetes [53]. There may, therefore, be common responses operating as a result of suboptimal nutrition in utero. There is a 50% reduction in the expression of *Cacna1c* and *Cacna1d*, which is key for GSIS, along with impairment of docking of insulin granules in male offspring of obese dams. The latter could be due to reduced ATP levels [54] owing to reduced ATP-linked respiration in these islets. Whilst not yet detrimental to GSIS at this age, these changes could mean that their islets are more vulnerable to metabolic insult/stress. This may partly explain why islets from maternal obesity-exposed male offspring that were subsequently exposed to a ‘second hit’ of high-fat feeding in postnatal life are more vulnerable to dysfunction compared with females [9]. Furthermore, our results highlight the role of pancreatic islets in conferring increased type 2 diabetes risk in male offspring exposed to maternal obesity.

Type 2 diabetes is associated with islet dysfunction and altered endocrine cell mass [55]. Whilst we found no difference in offspring beta/alpha cell mass, previous studies showed that maternal HFD impacted on offspring endocrine cell mass/number in neonatal and/or adult life (reviewed in [5]). Importantly, these studies also provided evidence for the dynamic nature of beta cell mass in these offspring and its ability to adapt to altered metabolic demand. Neonates exposed to maternal HFD throughout gestation have reduced beta cell number and volume at birth [56] but this difference disappears in adolescence [57]. Furthermore, exposure to maternal obesity led to reduced beta cell mass in 36-day-old offspring but this was higher at 110 days [11]. Thus, it is likely that differences between studies are due to differences in the age and metabolic status of the offspring at the time of investigation. Finally, it is also important to consider changes in beta cell mass in the context of its functional state since beta cells can occur under different phenotypes that vary with age and environmental conditions [58].

Looking to the future, it is important to improve our understanding of the possible maternal factors, e.g. hyperinsulinaemia, hyperglycaemia and hyperlipidaemia, underlying the programming of metabolic adversity across the life course of the offspring. Furthermore, it is also key that we identify the critical developmental period(s) during which suboptimal maternal nutrition programs altered islet function in the offspring.

In summary, the current findings suggest that sex differences in type 2 diabetes susceptibility in offspring as a consequence of exposure to maternal obesity could, at least in part, be driven by differences in islet function. Females, in response to nutritional cues from the mother signalling a nutrient-rich environment, prime islet development to thrive in this environment postnatally. In contrast, males may respond to these cues in a manner that minimises the risk of neonatal hypoglycaemia and maximises neonatal survival. However, this becomes maladaptive in later life following the onset of obesity and insulin resistance in these male offspring.

## Electronic supplementary material

ESM (PDF 1119 kb)

## Data Availability

The datasets generated during the current study are available from the corresponding author upon reasonable request.

## Abbreviations

ATP5A: ATP synthase F1 subunit α
A^vy^: Agouti viable yellow
BAX: Bcl-2-associated X protein
Bcl-2: B cell lymphoma 2
Ca_v_1: L-type voltage-gated Ca^2+^ (channel)
DCFDA: 2′,7′-dichlorofluorescin diacetate
ERα: Oestrogen receptor α
FCCP: Carbonyl cyanide-4-(trifluoromethoxy)phenylhydrazone
GSIS: Glucose-stimulated insulin secretion
HFD: High-fat diet
mtDNA: Mitochondrial DNA
ROS: Reactive oxygen species
STX1A: Syntaxin-1A
STXBP1: Syntaxin binding protein 1
TFAM: Mitochondrial transcription factor A
UQCRC2: Ubiquinol-cytochrome C reductase core protein 2
