# Supplementary material for: Exposure to maternal obesity programs sex differences in pancreatic islets of the offspring in mice
Source: Diabetologia. 2019 Nov 26;63(2):324–37. doi: 10.1007/s00125-019-05037-y (PMC6946752; doi:10.1007/s00125-019-05037-y)
Supplement: Supplementary file 1 — (PDF 1119 kb) [file 125_2019_5037_MOESM1_ESM.pdf]

ESM Table 1: Oligonucleotide primer sequences for qRT-PCR analysis

Gene Name (GenBank accession no.)	Forward	Reverse
Actb (NM_007393.5)	5’ GCCCTGAGGCTCTTTTCCAG3’	5’ TGCCACAGGATTCCATACCC3’
Hprt (NM_013556.2)	5’ GAGAGCGTTGGGCTTACCTC3’	5’ ATCGCTAATCACGACGCTGG3’
Rplp0 (NM_007475.5)	5’GAAAAGTTCTTGATCCCCAATGC3’	5’ TGTGACTGGTCCACAATTCCTT3’
mt-Nd5 (NC_005089.1)	5’ACGAAAATGACCCAGACCTC3’	5’ AGATGACAAATCCTGCAAAGATG3’
mt-Cytb (NC_005089.1)	5’CCCACCCCATATTAAACCCG3’	5’GAGGTATGAAGGAAAGGTATAAGGG3’
mt-Co1 (NC_005089.1)	5’TCCCAGATATAGCATTCCCACG3’	5’ACTGTTCATCCTGTTCCTGC3’
mt-Atp8 (NC_005089.1)	5’GCCACAACTAGATACATCAACATG3’	5’TGGTTGTTAGTGATTTTGGTGAAG3’
Sdha (NM_023281.1)	5’TTACAAAGTGCGGGTCGATGA3’	5’TGTTCCCCAAACGGCTTCTT3’
Cat (NM_009804.2)	5’AGCGACCAGATGAAGCAGTG3’	5’TCCGCTCTCTGTCAAAGTGTG3’

**Gene Name (GenBank accession no.)**	**Forward**	**Reverse**
Sod2 (NM_013671.3)	5’CAGACCTGCCTTACGACTATGG3’	5’CTCGGTGGCGTTGAGATTGTT3’
Casp3 (NM_001284409.1)	5’TGGTGATGAAGGGGTCATTTATG3’	5’TTCGGCTTTCCAGTCAGACTC3’
Casp9 (NM_001277932.1)	5’TCCTGGTACATCGAGACCTTG3’	5’AAGTCCCTTTCGCAGAAACAG3’

ESM Table 2: Antibodies used for Western blotting

Protein	Characteristic	Company and location	Catalogue No.	Dilution
Alexa Fluor® 488	Donkey anti-guinea pig	Jackson ImmunoResearch, Ely, Cambridgeshire, UK	705-545-148	1:100
ATP5A	Mouse monoclonal to Complex V subunit ATP5A (Total OXPHOS Rodent WB Antibody Cocktail)	Abcam, Cambridge, Cambridgeshire, UK	ab110413	1:1000
BAX	Rabbit polyclonal to Bax	Cell Signalling Technology, Danvers, MA, USA	#2772	1:1000
BCL-2	Rabbit monoclonal to BCL-2	Cell Signalling Technology	#3498	1:500
Cleaved caspase-3	Rabbit polyclonal to the large fragment of caspase-3 resulting from cleavage	Cell Signalling Technology	#9662	1:500
Cyanine Cy™5	Donkey anti-rabbit	Jackson ImmunoResearch	711-175-152	1:250
DAPI		Thermo Fisher Scientific, Carlsbad, CA, USA	D1306	1:2500 (5 mg/ml stock solution)
ERα	Mouse monoclonal to ERα	Santa Cruz Biotechnology, Dallas, TX, USA	sc-8002	1:500
GCG	Rabbit polyclonal to GCG	Bioss Antibodies, Woburn, MA, USA	bs-3796R	1:100

**Protein**	**Characteristic**	**Company and location**	**Catalogue No.**	**Dilution**
GK	Rabbit polyclonal to GK	Santa Cruz Biotechnology	sc-7908	1:500
GLUT2	Rabbit polyclonal to Glucose Transporter GLUT2	Abcam	ab54460	1:1000
INS	Guinea-pig polyclonal to INS	Dako, Technologies, Santa Clara, CA, USA	A0564	1:50
MT-CO1	Mouse monoclonal to Complex IV subunit MT-CO1 (Total OXPHOS Rodent WB Antibody Cocktail)	Abcam	ab110413	1:1000
NDUFB8	Mouse monoclonal to Complex I subunit NDUFB8 (Total OXPHOS Rodent WB Antibody Cocktail)	Abcam	ab110413	1:1000
SDHB	Mouse monoclonal to Complex II subunit SDHB (Total OXPHOS Rodent WB Antibody Cocktail)	Abcam	ab110413	1:1000
STX1A	Mouse monoclonal to STX1A	Synaptic Systems, Germany	110 111	1:1000
STXBP1	Rabbit polyclonal to STXBP1	Synaptic Systems	116 002	1:1000
TFAM	Rabbit polyclonal to TFAM	Abcam	ab47517	1:500

**Protein**	**Characteristic**	**Company and location**	**Catalogue No.**	**Dilution**
UQCRC2	Mouse monoclonal to Complex III subunit UQCRC2 (Total OXPHOS Rodent WB Antibody Cocktail)	Abcam	ab110413	1:1000
β-Tubulin	Rabbit polyclonal to β-Tubulin	Abcam	ab15568	1:5000

ESM Table 3: Body weight and serum glucose and insulin concentrations in male and female offspring of control and obese dams.

	Control		Obese	
	**Males**	**Females**	**Males**	**Females**
**Body weight (g)**	25.3 ± 0.7*	20.6 ± 0.4	25.3 ± 0.9*	20.5 ± 0.5
**Blood glucose concentration (mmol/l)**	13.2 ± 1.2	11.5 ± 0.8	12.7 ± 1.1	10.9 ± 0.5
**Serum insulin concentration (pmol/l)**	245 ± 31**	124 ± 17	257 ± 29**	166 ± 34

Blood was collected from fed animals at eight weeks of age. Data were analysed by two-way ANOVA followed by Tukey's multiple comparisons test. *P<0.05 and **P<0.01 for an effect of offspring sex. Males, *n*=8 mice/group and females, *n*=7 mice/group. '*n*' represents the number of mice from separate litters. All data are mean ± s.e.m.

ESM Fig. 1: Images of Western blots used for quantification of protein abundance

a.

Glucose transporter 2 (Females)

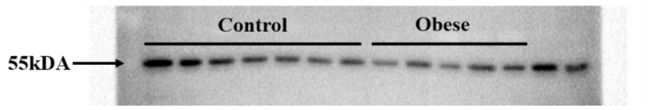

β-tubulin (Females)

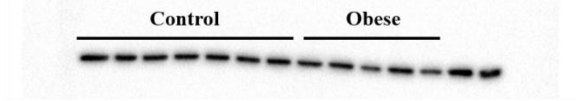

Glucose transporter 2 (Males)

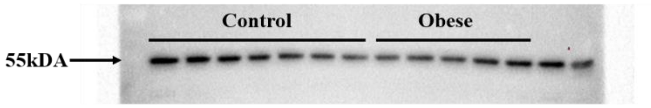

β-tubulin (Males)

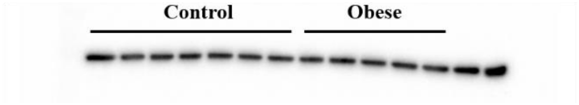

b.

Glucokinase (Females)

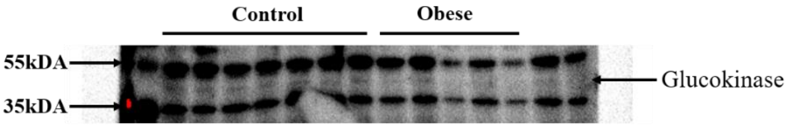

β-tubulin (Females)

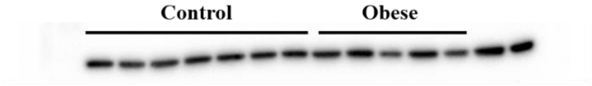

Glucokinase (Males)

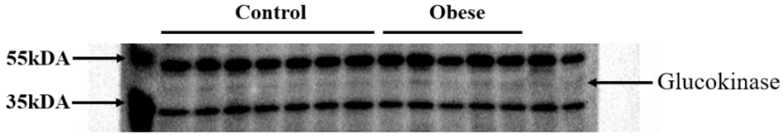

β-tubulin (Males)

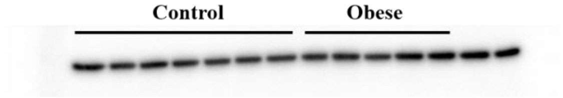

c.

Mitochondrial transcription factor A (Mitochondrial) (Females)

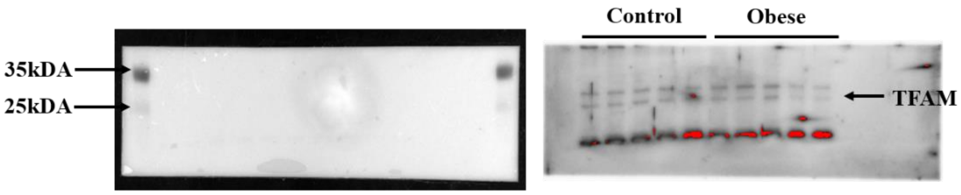

β-tubulin (Females)

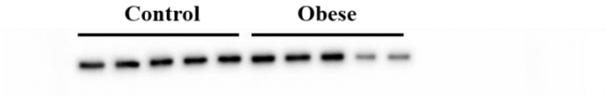

Mitochondrial transcription factor A (Mitochondrial) (Males)

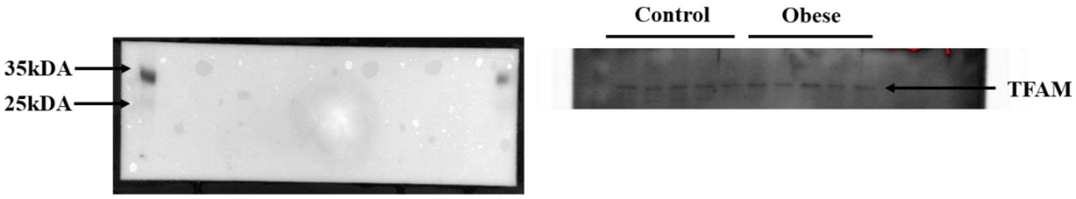

β-tubulin (Males)

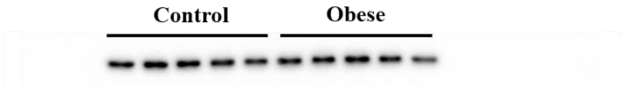

d.

Total OXPHOS (Complex I) (Females)

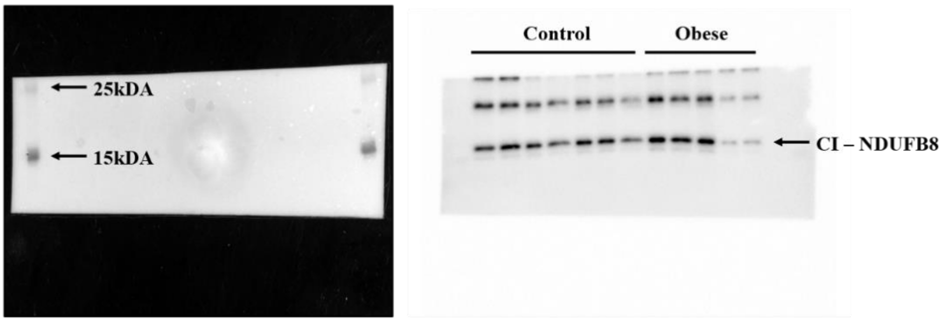

β-tubulin (Females)

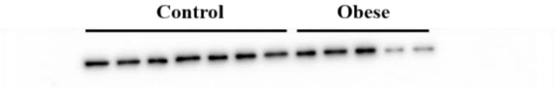

Total OXPHOS (Complex II to V) (Females)

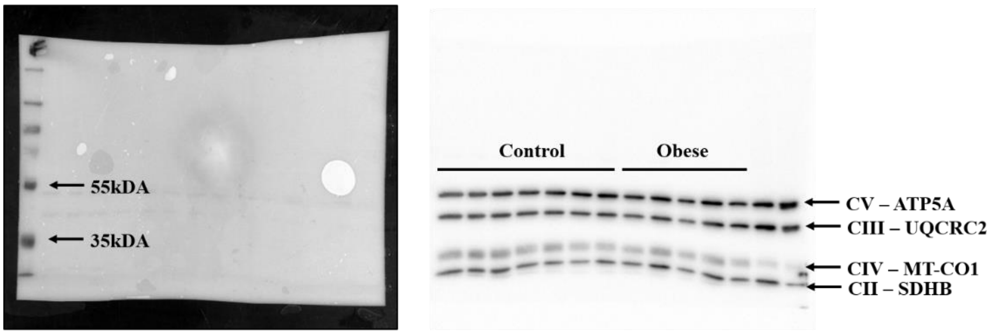

β-tubulin (Females)

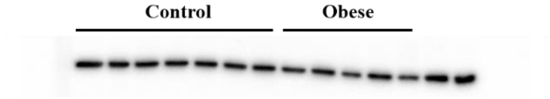

Total OXPHOS (Complex I) (Males)

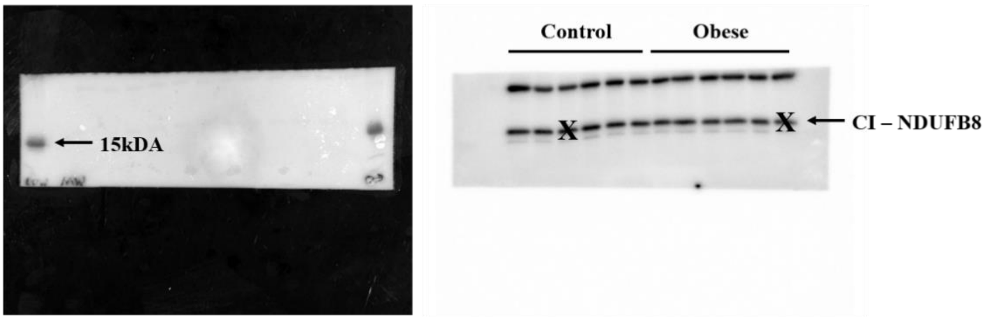

β-tubulin (Males)

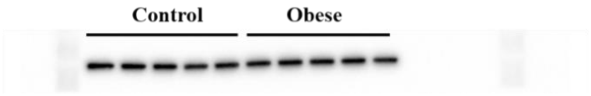

Total OXPHOS (Complex II to V) (Males)

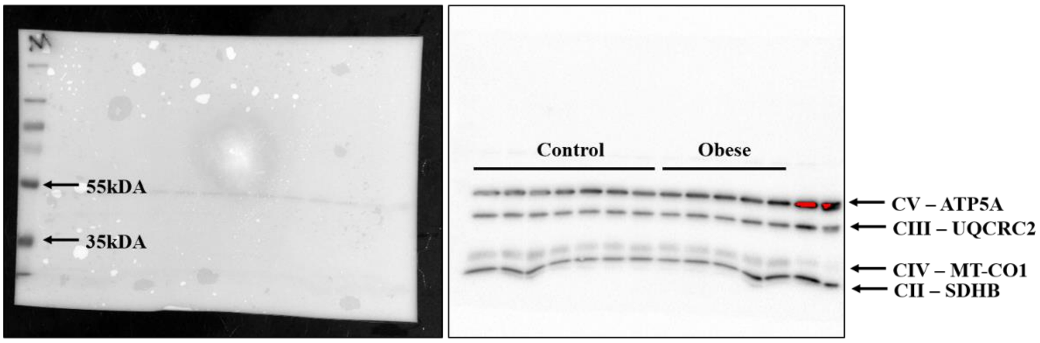

β-tubulin (Males)

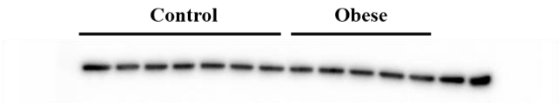

e.

Syntaxin 1a (Females)

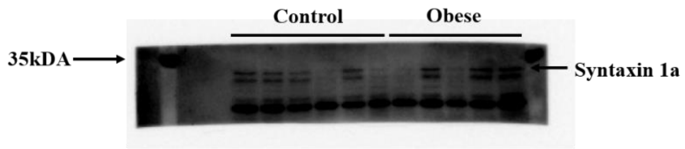

β-tubulin (Females)

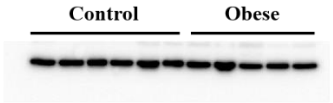

Syntaxin 1a (Males)

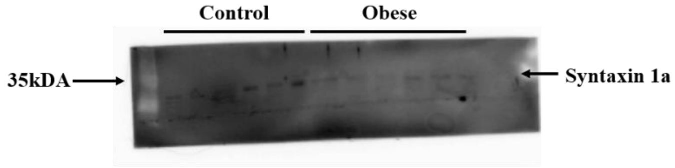

β-tubulin (Males)

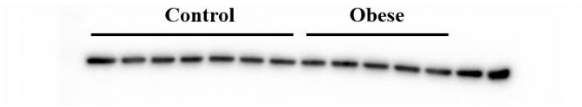

f.

Syntaxin binding protein 1a

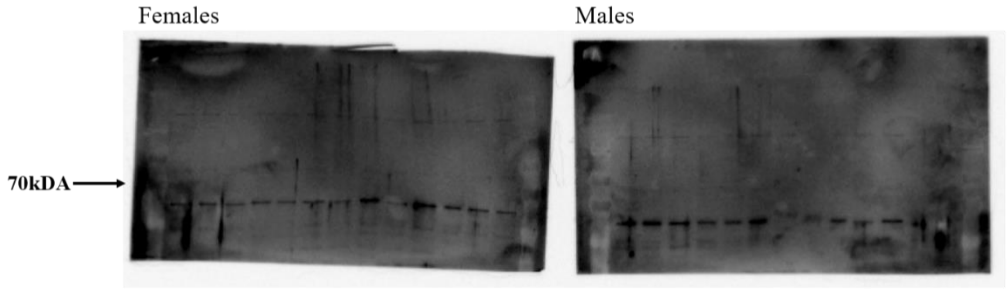

β-tubulin

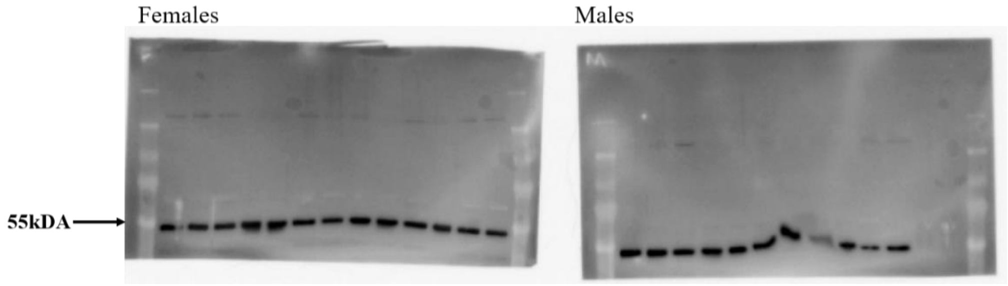

g.

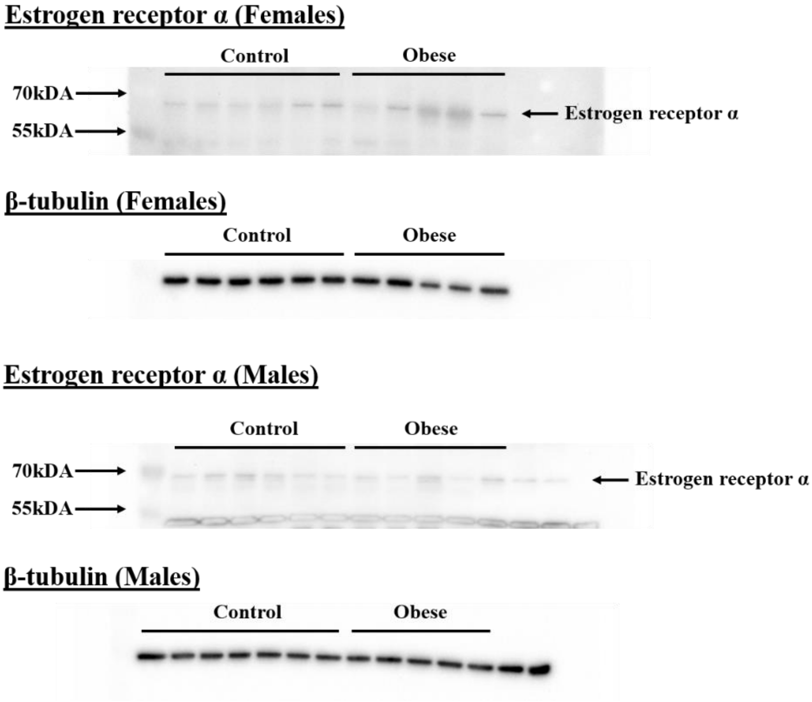
Estrogen receptor α (Females)
Control
Obese
70kDA
55kDA
Estrogen receptor α
β-tubulin (Females)
Control
Obese
Estrogen receptor α (Males)
Control
Obese
70kDA
55kDA
Estrogen receptor α
β-tubulin (Males)
Control
Obese

h.

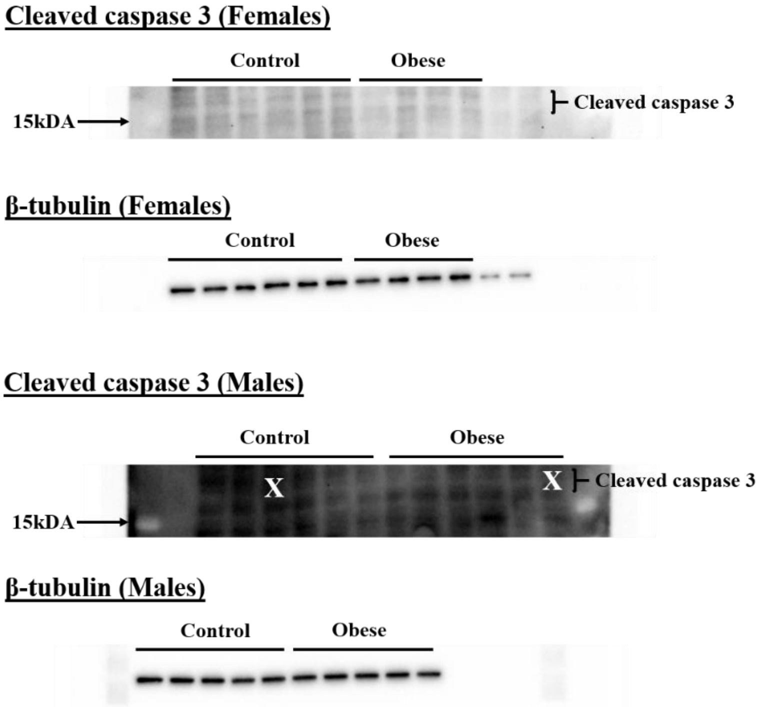
Cleaved caspase 3 (Females)
Control
Obese
15kDA
Cleaved caspase 3
β-tubulin (Females)
Control
Obese
Cleaved caspase 3 (Males)
Control
Obese
X
X
15kDA
Cleaved caspase 3
β-tubulin (Males)
Control
Obese

i.

Bax (Females)

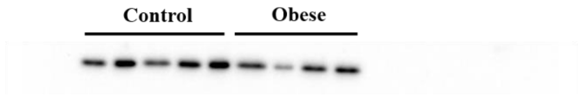

β-tubulin (Females)

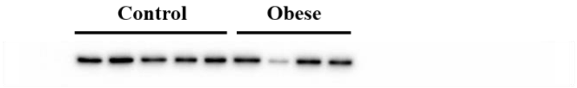

Bax (Males)

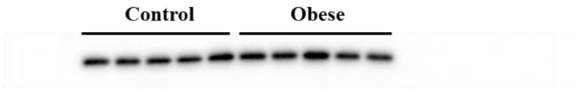

β-tubulin (Males)

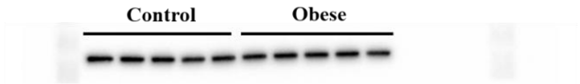

j.

Bcl-2 (Females)

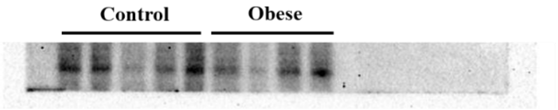

β-tubulin (Females)

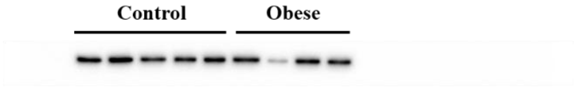

Bcl-2 (Males)

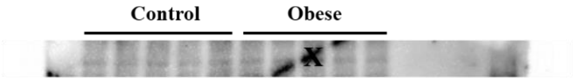

β-tubulin (Males)

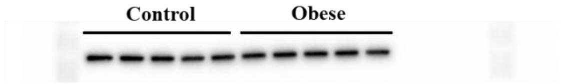

ESM Fig. 2: Male offspring had higher absolute pancreas weight and total pancreatic tissue area whilst female offspring had greater number of glucagon$^+$ cells, irrespective of maternal diet.

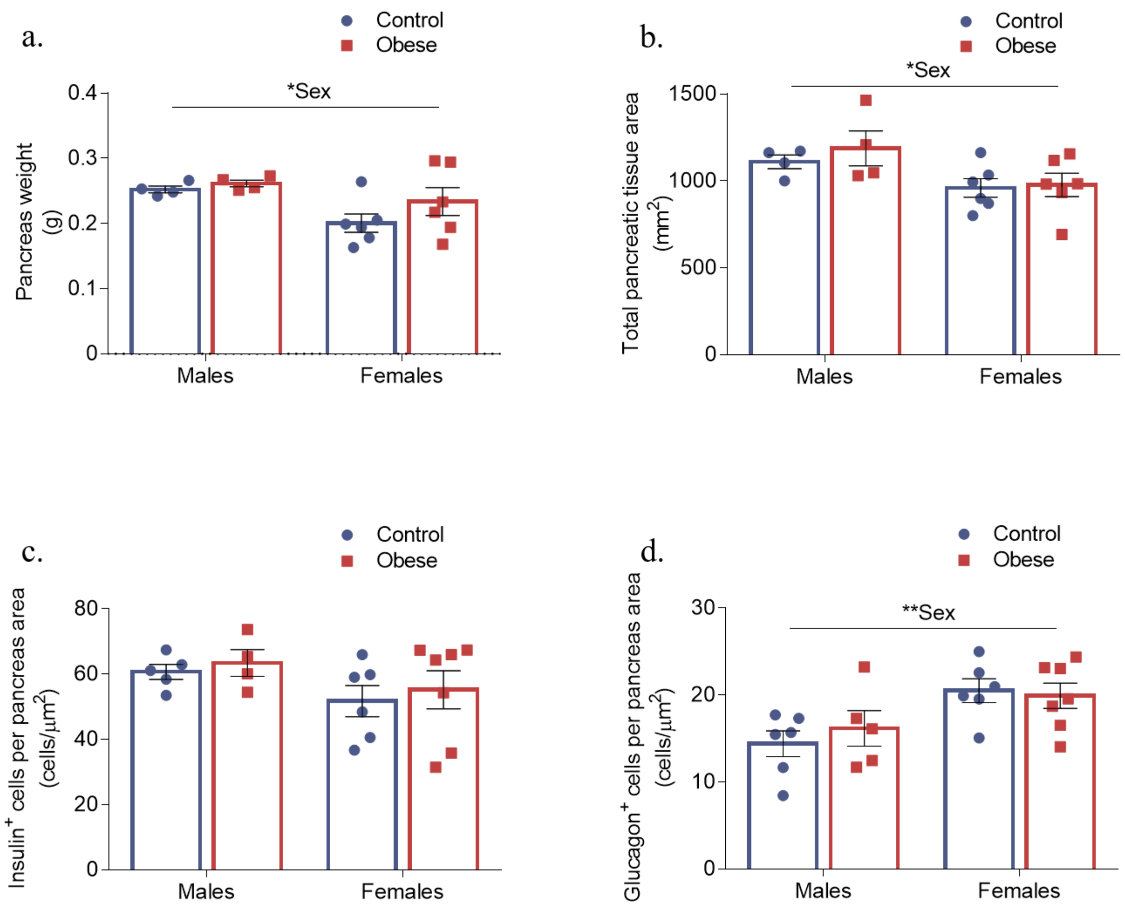

Absolute pancreas weight **(a)**, total pancreatic tissue area **(b)**, number of insulin$^+$ cells per pancreas area **(c)** and number of glucagon$^+$ cells per pancreas area in 8-week-old male and female offspring of control and obese dams. Data were analysed by two-way ANOVA. *p<0.05 and *p<0.01. (**a**, **b**) males, control: *n*=4, obese: *n*=4; females, control: *n*=6 obese: *n*=6 mice; (**c**) males, control: *n*=4, obese: *n*=4; females, control: *n*=6 obese: *n*=7 mice; (**d**) males, control: *n*=6, obese: *n*=5; females, control: *n*=6 obese: *n*=7 mice. ‘*n*’ represents the number of mice from separate litters. All data are mean ± s.e.m.

ESM Fig. 3: Exposure of male offspring to maternal obesity had minimal impact on mitochondrial DNA content and expression of mitochondrial and nuclear-encoded components of the electron transport chain.

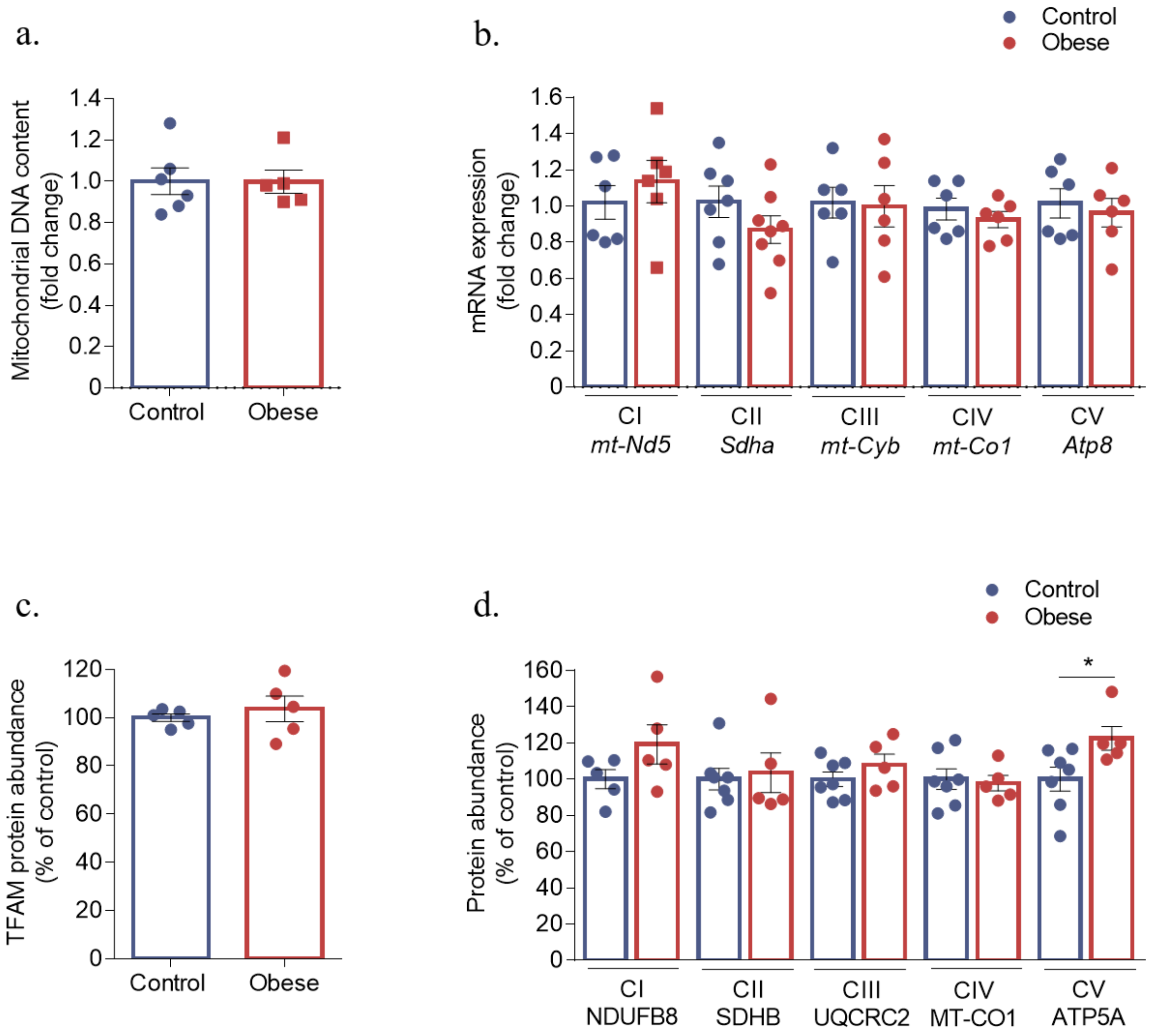

Mitochondrial DNA content **(a)**. qRT-PCR analysis of mRNA expression of mitochondrial (*mt-Nd5*, *mt-Cytb*, *mt-Co1*, *mt-Atp8*) and nuclear (*Sdha*) encoded components of the electron transport chain **(b)**. Western blot analysis of mitochondrial transcription factor A (TFAM) **(c)** and mitochondrial (MT-CO1) and nuclear (NFUFB8, SDHB, UQCRC2 and ATP5A) encoded components of the electron transport chain **(d)**. Image of corresponding Western blots: ESM Fig.1c (TFAM) and d (NFUFB8, SDHB, UQCRC2, MT-CO1 and ATP5A). Experiments were performed on islets isolated from eight-week-old male mice. Data were analysed independently

by unpaired Student’s t-test (Control versus Obese). *p<0.05. (**a**) control: *n*=6; obese: *n*=5 mice; (**b**) *mt-Nd5*, control: *n*=6, obese: *n*=6; *Sdha*, control: *n*=7 obese: *n*=8; *mt-Cytb,* control: *n*=6, obese: *n*=6; *mt-Co1*, control: *n*=6 obese: *n*=6; *Atp8*, control: *n*=6, obese: *n*=6 mice; (**c**) *n*=5 mice/group; (**d**) NDUFB8, control: *n*=5, obese: *n*=5; SDHB, UQCRC2, MT-CO1 and ATP5A, control: *n*=7, obese: *n*=5 mice. ‘*n*’ represents the number of mice from separate litters. All data are mean ± s.e.m.

ESM Fig. 4: Exposure to maternal obesity had no impact on the expression of pro-apoptotic Bax or anti-apoptotic Bcl-2.

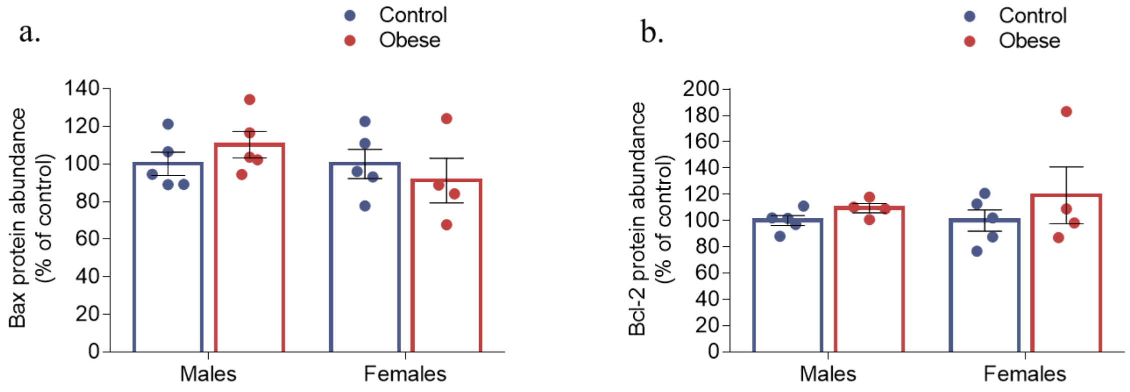

Western blot analysis of Bax **(a)** and Bcl-2 **(b)**. Image of corresponding Western blots: ESM Fig.1i (BAX) and j (Bcl-2). Experiments were performed on islets isolated from eight-week-old offspring. Male and female offspring were analysed independently by unpaired Student's t-test (control versus obese). (**a**) males, control: *n*=5, obese: *n*=5; females, control: *n*=5, obese: *n*=4 mice; (**b**) males, control: *n*=5, obese: *n*=4; females, control: *n*=5, obese: *n*=4 mice. '*n*' represents the number of mice from separate litters. All data are mean ± s.e.m.
